# A Preliminary Investigation of Radiation-Sensitive Ultrasound Contrast Agents for Photon Dosimetry

**DOI:** 10.3390/ph17050629

**Published:** 2024-05-14

**Authors:** Bram Carlier, Sophie V. Heymans, Sjoerd Nooijens, Gonzalo Collado-Lara, Yosra Toumia, Laurence Delombaerde, Gaio Paradossi, Jan D’hooge, Koen Van Den Abeele, Edmond Sterpin, Uwe Himmelreich

**Affiliations:** 1 Department of Oncology, KU Leuven-University of Leuven, 3000 Leuven, Belgium; bram.carlier@kuleuven.be (B.C.); laurence.delombaerde@kuleuven.be (L.D.); edmond.sterpin@kuleuven.be (E.S.); 2 Department of Imaging and Pathology, KU Leuven-University of Leuven, 3000 Leuven, Belgium; 3 Molecular Small Animal Imaging Center (MoSAIC), KU Leuven-University of Leuven, 3000 Leuven, Belgium; 4 Department of Physics, KU Leuven Campus Kortrijk—KULAK, Etienne Sabbelaan 53, 8500 Kortrijk, Belgium; sophie.heymans@kuleuven.be (S.V.H.); koen.vandenabeele@kuleuven.be (K.V.D.A.); 5 Department of Cardiovascular Sciences, KU Leuven-University of Leuven, 3000 Leuven, Belgium; sjoerd.nooijens@kuleuven.be (S.N.); jan.dhooge@kuleuven.be (J.D.); 6 Department of Cardiology, Erasmus MC University Medical Center, 3015 GD Rotterdam, The Netherlands; g.colladolara@erasmusmc.nl; 7 National Institute for Nuclear Physics, INFN Sezione di Roma Tor Vergata, 00133 Rome, Italy; yosra.toumia@uniroma2.it; 8 Department of Chemical Science and Technologies, University of Rome Tor Vergata, 00133 Rome, Italy; gaio.paradossi@uniroma2.it; 9 Department of Radiotherapy, UH Leuven, 3000 Leuven, Belgium; 10 Particle Therapy Interuniversity Center Leuven—PARTICLE, 3000 Leuven, Belgium

**Keywords:** radiotherapy, ultrasound imaging, phase-change contrast agent, nanodroplets, dosimetry

## Abstract

Radiotherapy treatment plans have become highly conformal, posing additional constraints on the accuracy of treatment delivery. Here, we explore the use of radiation-sensitive ultrasound contrast agents (superheated phase-change nanodroplets) as dosimetric radiation sensors. In a series of experiments, we irradiated perfluorobutane nanodroplets dispersed in gel phantoms at various temperatures and assessed the radiation-induced nanodroplet vaporization events using offline or online ultrasound imaging. At 25 °C and 37 °C, the nanodroplet response was only present at higher photon energies (≥10 MV) and limited to <2 vaporization events per cm^2^ per Gy. A strong response (~2000 vaporizations per cm^2^ per Gy) was observed at 65 °C, suggesting radiation-induced nucleation of the droplet core at a sufficiently high degree of superheat. These results emphasize the need for alternative nanodroplet formulations, with a more volatile perfluorocarbon core, to enable in vivo photon dosimetry. The current nanodroplet formulation carries potential as an innovative gel dosimeter if an appropriate gel matrix can be found to ensure reproducibility. Eventually, the proposed technology might unlock unprecedented temporal and spatial resolution in image-based dosimetry, thanks to the combination of high-frame-rate ultrasound imaging and the detection of individual vaporization events, thereby addressing some of the burning challenges of new radiotherapy innovations.

## 1. Introduction

Radiotherapy is one of cancer’s primary treatment options, received by approximately 50% of patients with solid tumors [1]. The vast majority of patients are treated with external beam photon radiotherapy. State-of-the-art treatment delivery features image-guided radiotherapy (IGRT) and intensity-modulated radiotherapy (IMRT), often associated with continuous rotation of the gantry (volumetric arc therapy (VMAT)), which yields highly conformal treatments [2]. Yet, the associated steep dose gradients have imposed additional requirements on the treatment quality assurance [3].

In vivo dosimetry is one important but often underexplored part of this quality assurance chain, whereby the radiation dose received by the patient is assessed during irradiation [3,4]. Historically, a plethora of in vivo dosimeters have been used, which can be classified based on the dimensionality of their read-out [3]. Point dosimeters comprise diodes, metal-oxide semi-conductor field effect transistors (MOSFETs), plastic scintillation detectors (PSD), optically stimulated luminescent dosimeters (OSLDs), thermoluminescent dosimeters (TLDs), and radiophotoluminescent dosimeters (RPLDs), and provide measurements of the dose at a single point. While often well-characterized, these dosimeters provide limited information to validate more complex treatment plans. Two-dimensional dosimeters include radiographic and radiochromic film. They are able to provide 2D dose distributions with high spatial resolution. However, the complex read-out makes their use cumbersome and prevents real-time dose assessment. Therefore, clinically relevant efforts are primarily directed at electronic portal imaging devices (EPIDs) [4]. These systems make use of a flat panel detector including a scintillating layer and an array of photodiodes to determine the transmitted X-ray dose. Through forward- or back-projection methods, this information can then be used to verify the treatment delivery inside the patient in three dimensions (3D) [5,6]. However, the existing vendor–user gap, non-water equivalence of the detector, and the difficulty in defining meaningful parameters to identify when a treatment plan is compromised have slowed down clinical implementation [4,7]. As a result, alternative in vivo dosimetry solutions are still actively being sought.

About a decade ago, Verboven et al. proposed a radically different dosimetry approach based on ultrasound contrast agents [8]. They postulated that ionizing radiation is capable of changing the ultrasonic properties of microbubbles from which the radiation dose can be deduced. Initial investigations illustrated a dose-dependent attenuation behavior of the Targestar-P microbubble formulation. Unfortunately, the production of Targestar-P was discontinued shortly afterwards, and reproduction of these results with other commercially available microbubbles has only shown very modest results [9]. More promising is the recent development of superheated phase-change nanodroplets [10,11,12]. These particles, consisting of a perfluorocarbon core and a lipidic, polymeric, or protein shell, can undergo a phase transition from the liquid to gas state upon external stimulation [13,14]. Such stimuli include high-pressure ultrasound waves (acoustic droplet vaporization, ADV) [15], laser heating (optical droplet vaporization, ODV) [16], magnetic stimulation (magnetic droplet vaporization, MDV) [17], and most interesting in our context, radiation-induced vaporization (RIDV) [18]. The latter case is described by the theory of radiation-induced nucleation of superheated emulsions, explaining that a charged particle depositing sufficient energy over a certain characteristic length (i.e., having a sufficiently large linear energy transfer (LET)) is able to nucleate the superheated droplet into a microbubble [19,20]. This phase transition corresponds to the sudden generation of strong ultrasound contrast, which can be used to assess radiation delivery. While primarily investigated for proton range verification [18,21,22], a preliminary investigation into photon beams also demonstrated their potential use for photon dosimetry [11].

In this contribution, we elaborate initial findings by investigating the potential mechanisms by which photons induce droplet vaporization. For this purpose, gel phantoms containing different nanodroplet formulations were irradiated at varying temperatures and the resulting observations were related to the previously described theoretical framework of radiation-induced nucleation of superheated emulsions [19]. In addition, we explored different ultrasonic read-outs (offline vs. online) for the detection and follow-up of the radiation response and illustrated the critical role of the phantom matrix to establish reproducible dose–response relationships. Finally, we discuss challenges and opportunities of the described technology and make suggestions on how to tackle these challenges to facilitate eventual clinical implementation.

## 2. Results

### 2.1. Radiation-Induced Nucleation of the Superheated Nanodroplet Core Is the Primary Mechanism of Ultrasound Contrast Generation by Photon Beams

**Figure 1:**
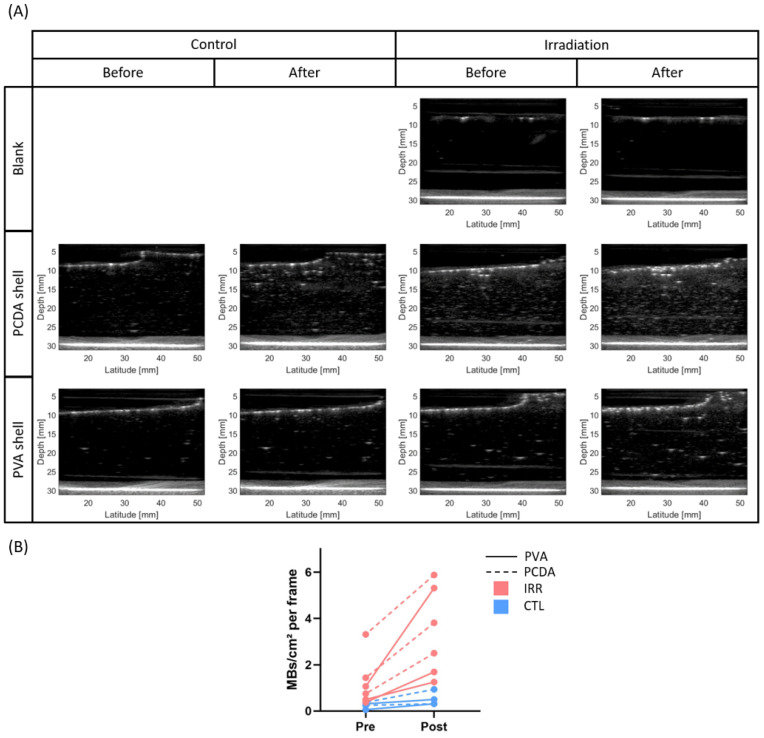
Offline nanodroplet photon response at 25 °C. (**A**) Gelatine phantoms containing no nanodroplets (blank), 25 µM of PCDA-PFB nanodroplets (PCDA shell), and 25 µM of PVA-PFB nanodroplets (PVA shell). Corresponding images are shown before and after irradiation with 10 Gy photons (10 MV) at 2 Gy/min (irradiation) or incubation in a water tank under the same conditions as the irradiated phantoms (control). (**B**) Microbubble (MB) quantification in a 2 × 1 cm ROI for 8 frames across each phantom. CTL = control phantoms (n = 2), IRR = irradiated phantoms (n = 3).

To investigate the potential mechanisms through which photon beams generate ultrasound contrast, two types of nanodroplets were homogeneously dispersed in gel phantoms and subsequently irradiated with a clinical photon beam at 25 °C. One formulation consisted of a crosslinked monolayer lipidic shell (10,12-pentacosadyinoic acid—perfluorobutane, PCDA-PFB), while the other comprised a polymeric shell (poly(vinyl alcohol)—perfluorobutane, PVA-PFB). Figure 1 shows representative ultrasound images before and after irradiation (or submersion in the control water bath) for the different nanodroplets. In addition, a blank without nanodroplets is shown to ensure that no signal artifacts derive from the gel. Despite the relatively high radiation dose of 10 Gy, only a mild response (0–0.5 vaporization events (microbubbles, MBs) per cm^2^ per frame per Gy) was observed for either nanodroplet formulation. A minimal increase in signal and microbubble formation was observed in post images of control phantoms (<1 MB/cm^2^ per frame). This could be due to the introduction of spontaneous vaporization events upon phantom preparation. Over time, these signals can increase due to bubble inflation, whereby perfluorocarbon from surrounding nanodroplets diffuses to the already formed microbubbles [23]. In addition, a slightly higher degree of background signal (~1–2 MBs/cm^2^ per frame, Figure 1B) was observed in the phantoms containing PCDA-PFB NDs, which can be explained by their limited stability. Indeed, we reported earlier that PCDA-PFB NDs exhibit inferior stability with respect to PVA-shelled counterparts [21].

To ensure that the limited contrast generation was due to a limited radiation response instead of a lack of vaporizable droplets, one of the irradiated PVA-PFB phantoms was exposed to high-pressure ultrasound waves after irradiation, which resulted in the formation of a clear vaporization zone (Figure S1 in Supplementary Materials). This confirmed that vaporizable droplets were present but were not triggered by the photon beam.

**Figure 2:**
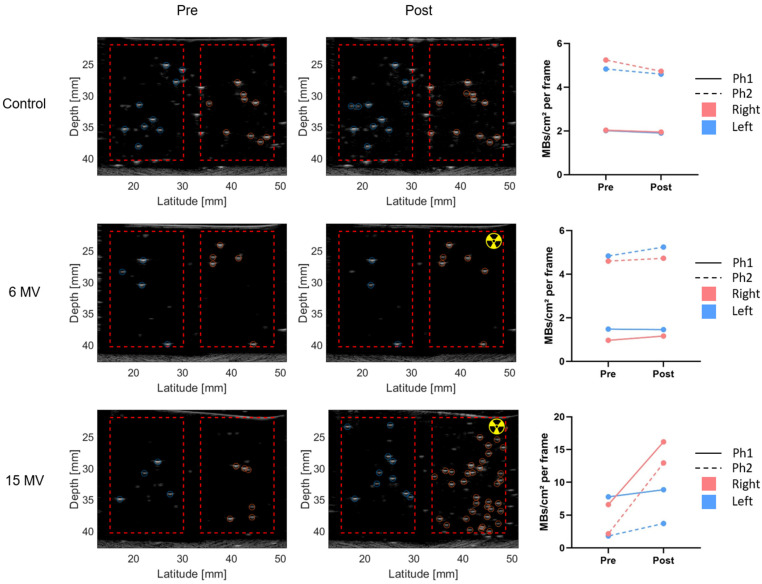
Offline nanodroplet photon response at 37 °C. Representative pre- and post-irradiation (or submersion) images of gel phantoms containing 50 µM PVA-PFB nanodroplets. Only the right-hand side of the phantom was placed inside the radiation field (10 × 10 cm) and was irradiated with a dose of 10 Gy for varying photon energies (radioactivity sign). Red dashed boxes indicate the ROIs in which microbubbles were counted. The right panel shows microbubble quantification in the left and right ROI of 17 frames across each phantom (n = 2, Ph#). Control phantoms were not irradiated but incubated in a water tank under the same conditions as the irradiated phantoms.

In a subsequent experiment, we increased the operation temperature to 37 °C. At a beam energy of 6 MV, the radiation response was minimal; however, at 15 MV, a clear radiation response was observed, which was more pronounced in the part of the phantom that was placed inside the radiation field (Figure 2). The small increase in microbubble formation in the non-irradiated region of interest (ROI), can be explained by analyzing the lateral profile of the beam, as elaborated in Section 4, illustrating that doses up to ~10% of the target dose are still obtained outside the radiation field. Yet, the radiation response remained minor even inside the radiation field with less than two vaporization events per cm^2^ per Gy.

**Figure 3:**
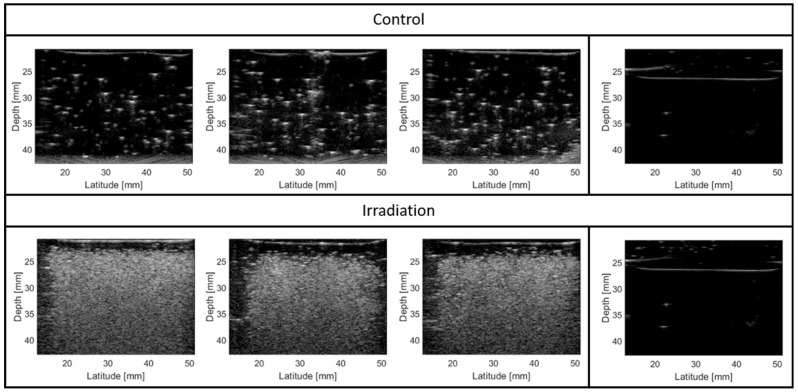
Offline nanodroplet photon response at 65 °C. Gel phantoms containing 25 µM of PVA-PFB nanodroplets. Ultrasound images of 3 different phantoms are shown after irradiation with 10 Gy photons (6 MV) at 4 Gy/min (irradiation) or incubation in a water tank under the same conditions as the irradiated phantoms (control). Of note is that only the right half of the phantom was placed in the radiation field (10 × 10 cm). On the right, a polyacrylamide phantom without nanodroplets before and after irradiation with 4 Gy photons (6 MV) at 4 Gy/min is shown to illustrate the lack of radiation response of the gel itself.

Finally, the operation temperature was further increased to 65 °C, a temperature where sensitization of the perfluorobutane core to the (secondary electrons released by) the primary photon beam is expected, while the rest of the irradiation conditions were kept the same. At such high temperature, the background signals increased due to spontaneous droplet vaporization events, yet remained limited (Figure 3). More importantly, an obvious increase in ultrasound contrast was observed after irradiation, but it remained, however, homogenous throughout the entire phantom despite the fact that only the right half was placed inside the radiation field. This suggests that the signal was completely saturated, even for the left-hand side, where doses of ~10% at most are deposited.

### 2.2. Online Ultrasound Imaging Allows Capturing the Dynamics of the Radiation Response by Detecting Individual Vaporization Events over Time

**Figure 4:**
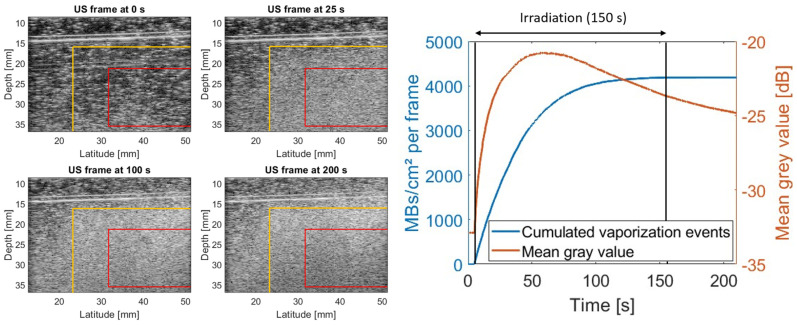
Online nanodroplet radiation response at 65 °C. Ultrasound frames of a carbopol gel phantom containing 40 µM PVA-PFB NDs at different time points during irradiation with 2 Gy photons (6 MV) at a dose rate of 0.8 Gy/min. Yellow lines indicate the edges of the radiation field (50% isodose line). Red box indicates the ROI used for the signal quantification displayed on the right.

The observed signal saturation also highlighted a key weakness of the used imaging approach that does not capture the dynamics of contrast generation. Therefore, we transitioned towards an online imaging approach in the following experiments, inspired by Collado-Lara et al. [22]. While offline images do not allow quantifying individual vaporization events in dense bubble clouds, ultrasound imaging at sufficiently high frame rates enables us to detect and localize vaporization events in the differential images. This way, we could assess signal generation both via conventional analysis of the mean gray value in the designated ROI as well as by counting the amount of vaporizations taking place. An example of such online evaluation of signal generation is depicted in Figure 4 (exact irradiation conditions listed in Table 1, exp. 3, row 2). At the start of the irradiation, nanodroplet vaporization takes primarily place within the radiation field (yellow boundaries). However, the signal saturates rapidly inside the ROI, while vaporization events outside of the radiation field still increase over time. As the radiation dose (2 Gy) was only a fraction of the one used in Figure 3 (10 Gy), this supports our previous hypothesis of signal saturation also outside the radiation field. Interestingly, the comparison between mean gray value analysis and counting of individual vaporization events indicates that the average gray value more rapidly saturates, and in this case, even drops near the end of the irradiation due to acoustic attenuation by the microbubble cloud above the ROI. This emphasizes the limitations of conventional gray value analysis for potential dosimetric applications, especially considering that the gray value signals kept changing even after irradiation had ceased. The latter could again be explained by bubble inflation. While counting individual microbubbles on differential frames allowed us to detect new vaporization events over the whole course of the irradiation, a decrease in the vaporization rate was also observed. Nevertheless, signal generation only occurred during irradiation and did not alter afterwards, making it more reliable to identify correlations with the photon dose. Noteworthy is that the amount of microbubbles counted per cm^2^ (~2000 per Gy) was about three orders of magnitude larger than the responses observed at 25 °C and 37 °C.

### 2.3. Use of the Suboptimal Carbopol Phantom Matrix Prevented Quantification of Reproducible Dose–Response Relationships

**Figure 5:**
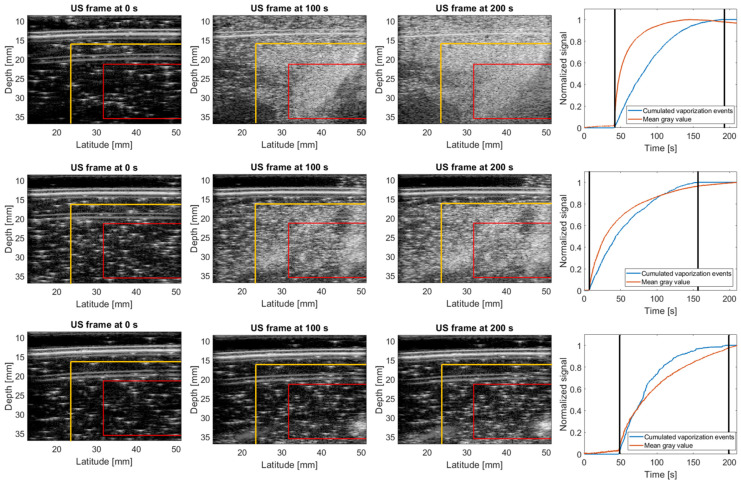
Reproducibility of the nanodroplet photon radiation response at 65 °C. Ultrasound frames of a carbopol phantom containing 20 µM PVA-PFB NDs at different time points during irradiation with 6 Gy photons (6 MV) at a dose rate of 2.4 Gy/min. Yellow lines indicate the edges of the radiation field (50% isodose line). Red box indicates the ROI used for the signal quantification displayed on the right, where the black vertical lines indicate the start and end of the irradiation.

To examine the reproducibility of signal generation, six phantoms were irradiated under the exact same conditions. The results of three of these phantoms are summarized in Figure 5 to cover the variety of responses observed (strong (row 1)—partial (row 2)—minimal (row 3)). While the first phantom exhibited a response similar to the one shown in Figure 4, some phantoms barely provided a radiation response except for very localized spots. This could potentially be explained by the used phantom matrix. In the first and second experiment shown in Table 1, droplets were dispersed in the respective gel matrices before gelation. The carbopol matrix used in this experiment did not transition from a liquid to a gel phase, but continuously exhibited a non-Newtonian behavior able to trap the nanodroplets. While droplets were extensively mixed in the gel matrix with a spatula, it appears that the homogeneity of the distribution was suboptimal. Even in the phantoms where a strong or partial radiation response was observed (row 1 and 2 in Figure 5), zones of higher and lower microbubble density were present, supporting this explanation. Despite the variable response, similar conclusions can be drawn with respect to the signal quantification, as described before. For the phantoms with dense microbubble clouds, evaluation based on the mean gray value resulted in faster signal saturation. Moreover, the grey value signal also slightly changed before and after irradiation, while an increase in detected vaporization events only occurred during irradiation. In phantoms with a less pronounced radiation response, both types of analysis followed a similar behavior.

### 2.4. Phase-Change Ultrasound Contrast Agents Induce Concentration-Dependent Magnetic Resonance Contrast after Droplet Vaporization

**Figure 6:**
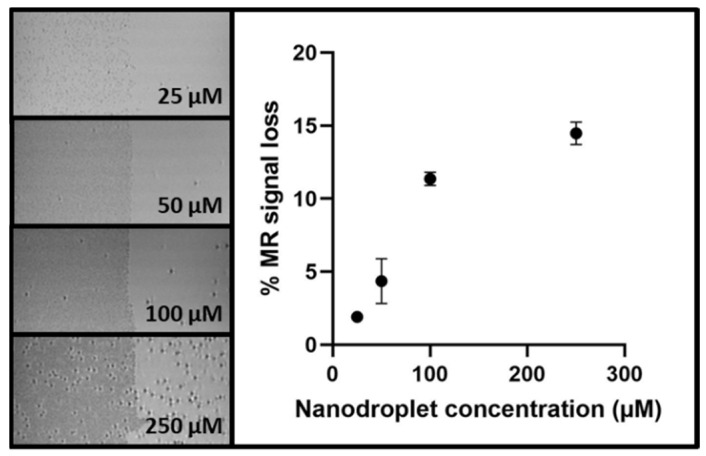
Concentration-dependent generation of negative MR contrast after acoustic droplet vaporization of PVA-PFB nanodroplet-containing gelatine phantoms. Acoustic vaporization was limited to the left half of the phantom to assess signal loss with respect to the non-vaporized right half. Average signal loss and corresponding standard deviations are displayed on the right (n = 3).

The typically limited field of view in ultrasound imaging might potentially complicate the evaluation of complex 3D treatment plans. Therefore, in a final experiment, we explored the multimodal contrast generation in magnetic resonance (MR) images. For this purpose, droplet vaporization was induced acoustically in one-half of droplet-containing gel phantoms. Figure 6 illustrates how the generation of microbubble clouds results in loss of the MR signal intensity with respect to the non-vaporized half of the phantom. Moreover, a quantitative relationship between the signal loss and nanodroplet concentration was identified which appeared to saturate at elevated concentrations. These results indicate that the proposed nanodroplet-mediated sensor is not restricted to ultrasonic read-outs, expanding its potential applications.

## 3. Discussion

### 3.1. Mechanisms of Photon-Induced Nanodroplet-Mediated Contrast Generation

We have investigated the potential of superheated nanodroplets for ultrasound-based photon dosimetry. First, we explored the potential mechanism through which the contrast generation occurs during irradiation, as X-rays can interact in different ways with the developed contrast agents. For example, Verboven et al. hypothesized several pathways through which ionizing radiation can destabilize the lipidic shell of microbubbles, including chemical modification due to free radicals generated by water radiolysis, direct absorption of radiation by the shell, and radiation-induced hydrolysis of the lipids [8]. Given the metastable state of the nanodroplets, it is not unlikely that disintegration of the shell could contribute to droplet vaporization. Alternatively, superheated emulsions can be vaporized if charged particles deposit sufficient energy over a certain characteristic length. The exact nucleation conditions have been described before [19] and seem to hold for superheated nanodroplets [18,21]. However, the secondary electrons released by photon irradiation are low LET radiation, consequentially requiring elevated temperatures (>60 °C) to sensitize the nanodroplets’ perfluorobutane core to photon irradiation. To be able to distinguish the potential contribution of both mechanisms, first, experiments were performed at temperatures below the photon sensitization of the perfluorobutane core.

As a radiation response was lacking at 37 °C for 6 MV photons, a potential radiation-induced disintegration of the nanodroplet shell and consecutive vaporization does not seem to significantly contribute to phase-change events. By changing the photon energy at 37 °C to 15 MeV, a (modest) radiation response was observed, which alludes to the effect of photoneutrons arising from interactions between the photons and the nuclei of high-Z materials in the treatment head (e.g., tungsten and copper) [24]. This also explains the limited radiation response at 25 °C, where a photon energy of 10 MV was used. Moreover, these findings are in agreement with observations made previously in proton beams, where a radiation response can be observed as a result of high LET secondary reaction products, even without sensitization to the primary particle beam [18,21]. Nevertheless, both at 25 °C and 37 °C, the radiation response was small with respect to the strong contrast generation during acoustic droplet vaporization after irradiation (Figure S1 in the Supplementary Materials) and not suitable for actual dosimetry. More interesting was the radiation response at 65 °C (with 6 MV photons), where microbubble counts of about three orders of magnitude larger than the results at lower temperature were detected. As control phantoms had limited background signals and no photoneutrons are generated at 6 MV, this large discrepancy in signal generation can only be explained by the sensitization of the superheated nanodroplet core to the photon beam.

The latter is indeed supported by the theory of radiation-induced nucleation of superheated emulsions that combines Seitz’s thermal spike theory [25] and the thermodynamics of homogenous nucleation [26]. This theory has been described extensively before [19], and is summarized in Equations (A1)–(A7) in Appendix A. It explains that the ionizing radiation needs to deposit a certain energy over a characteristic length to overcome the nucleation barrier. As a result, a vaporization threshold (*Vt*, Equation (A6)) can be calculated which describes the LET charged particles needed to satisfy the nucleation condition [18]. The thermodynamic parameters required to perform these calculations are provided in Table A1 in the Appendix A for perfluorobutane (PFB) at the different test temperatures. This results in a vaporization threshold of ~369 keV/µm at 25 °C, ~147 keV/µm at 37 °C, and eventually, ~21.8 keV/µm at 65 °C. Note that these values are approximate, as measurement errors on the reported quantities in Table A1 (Appendix A) exist. Nevertheless, these vaporization thresholds allow us to explain our observations as the secondary electrons produced by photon beams maximally reach around 26 keV/µm at the very end of their range [27]. As a result, for the investigated nanodroplet formulation, only at 65 °C are they able to trigger droplet vaporizations.

An additional parameter to describe and compare the sensitization of superheated liquids to different types of ionizing radiation is the reduced superheat (*s*, Equation (A7)) [19]. D’Errico identified for a range of different perfluorocarbons that photon sensitization starts occurring from ~*s* = 0.52; however, the strongest responses are seen for *s* > 0.60 [28]. This again agrees with our observations and partially explains why we observed significant variability in our results. Indeed, small temperature fluctuations (and thus changes in *s*) can signify the difference between an extensive or minimal radiation response. Analogously, an *s* > 0.33 was described by d’Errico to sensitize several halocarbons to thermal neutrons. Under the assumption that this statement holds for perfluorobutane, photoneutrons are indeed able to trigger droplet vaporization events at 37 °C, explaining why we observed an increased radiation response by switching from 6 to 15 MV at 37 °C. Furthermore, this agreement between theory and the experimental results again confirms that radiation-induced vaporization of the droplet core is the primary mechanism of ultrasound contrast generation.

### 3.2. Towards an In Vivo Application of Nanodroplet-Mediated Dosimetry

However, this also restricts the current nanodroplet formulation for in vivo applications. One way to overcome this issue is by replacing the nanodroplet core with a lower-boiling-point perfluorocarbon, which would decrease the vaporization threshold at 37 °C. Two examples are provided in Table A1 (Appendix A): heptafluoropropane (HFP) and octafluoropropane (OFP). From these two, OFP seems the most promising, as the vaporization threshold drops below the electron LET and nanodroplets constituted of an OFP core have been reported before [29,30,31]. However, d’Errico described that superheated perfluorocarbons start to spontaneously vaporize from a reduced superheat of 0.65 onwards [19], which would predict a lack of stability at body temperature. The HFP core, on the other hand, is not expected to exhibit photon sensitization at 37 °C. Hence, a solution might be in the mixing of different perfluorocarbons, an approach used before to alter the required pressures for acoustic droplet vaporization [32,33,34]. Either way, a delicate balance will have to be found between sufficient metastability to ensure photon sensitization on one hand and sufficient stability to allow their handling on the other. Another solution to sensitize the currently used nanodroplets to photon beams is by combining irradiation with an additional acoustic stimulus. A proof-of-concept of such an approach with proton irradiation has recently been published [35], but comes with additional uncertainties regarding the homogeneity of the applied acoustic field. Nevertheless, if the nanodroplet formulation can be sensitized to the photon beam at body temperature, in vivo dosimetry should be feasible. Indeed, we previously demonstrated an in vivo proof-of-concept of proton range verification with radiation-sensitive nanodroplets [36].

Furthermore, an in vivo application will require better control on the nanodroplet size distribution to ensure reproducibility and prevent potential biohazards. Currently, highly polydisperse samples were obtained due to the sonication-based production method. While size selection is possible using, for example, differential centrifugation [37], such an approach was logistically difficult to apply on the lab-scale production to ensure sufficient nanodroplet yield. Once the technology matures towards industrial-scale production, more monodisperse samples with strict size control will be required.

### 3.3. Towards Nanodroplet-Mediated Gel Dosimetry

Given the challenges towards an in vivo application, it is currently more realistic to develop the presented nanodroplet formulation towards an innovative type of gel dosimeter. One key limitation of conventional gel dosimeters (e.g., Fricke and polymer gels) is that the radiation response is only evaluated after irradiation [38,39]. Our approach can overcome this problem by employing high-frame-rate ultrasound imaging to detect and localize individual vaporization events during irradiation. While, in our current setup, only a modest frame rate of 10 Hz was achieved, given technical limitations of the scanner, examples of plane wave scanners operating at frame rates of 1–10 s of kHz have been described in the literature [22,40]. Moreover, using ultrasound localization microscopy to position individual microbubbles allows us to break through the diffraction limit, with achievable resolutions on the µm level [41,42].

To achieve this potential will, in the first place, require us to establish reproducible dose–response relationships. So far, this has been unsuccessful due to the suboptimal choice of the phantom matrix, resulting in excessive inter-phantom variability. Once the reproducibility issue is overcome, the same experiments should be repeated for a variety of radiation doses, dose rates, and radiation fields, to determine the robustness (or dependencies) of the proposed dosimeter. Afterwards, dose calibration curves can be determined based on the detected number of vaporization events. A similar simplified approach based on the visual/optical detection of millimeter-sized bubbles was previously commercialized for neutron dosimetry [43].

Another important characteristic of gel dosimeters is their dynamic range. Here, we observed that nanodroplet concentrations of a few tens of µM can result in complete saturation of the ultrasound contrast. This could be due to saturation of the ultrasound system itself, or the rapid diffusion of the perfluorobutane core, resulting in a decrease in vaporizable droplets. The observed saturation was partially overcome by transitioning to online ultrasound read-outs, allowing us to better quantify the radiation response. Individual detection and accumulation of vaporization events outperformed conventional gray value analysis both in terms of quantitative information (e.g., only signal generation during irradiation) and speed of saturation. However, even the employed individual detection had limitations and relied on the setting of an empirical gray value threshold. To overcome this issue, an alternative bubble detection method has been proposed based on deep learning methods (BubbleNet) [44]. For similar data, such an approach was able to detect up to 30% more vaporization events and proved to be robust across different experimental setups. In addition, future work will have to elucidate how varying the nanodroplet concentration influences signal saturation.

A final limitation of this study is the 2D ultrasound read-out. Given the often complex 3D nature of treatment plans, ultrasound acquisitions should ideally be acquired in 3D. For the first and second experiment, where ultrasound images were acquired offline, the ultrasound probe was moved laterally on a linear stage to acquire images across the entire phantom. By stacking the US images, a 3D read-out can be obtained, albeit with a resolution in the sagittal and coronal plane limited by the step size used (Figure S2). While motorized motion of a 2D linear probe can allow online imaging in 3D, more promising is the ongoing development of matrix probes that can image directly in 3D [45,46]. Alternatively, we can also harness the contrast generated by the microbubble clouds in different imaging modalities to obtain 3D information with high spatial resolution. For example, we were able to identify a concentration-dependent loss in magnetic resonance (MR) signal after acoustic droplet vaporization of nanodroplets. This shows that our dosimeter is compatible with different imaging modalities. Careful optimization of the nanodroplet concentration and the desired read-out time will, however, be required. Rapid saturation of the MR signal was observed at elevated concentrations attributed to the nature of negative contrast (signal intensity cannot drop below zero) and the phenomenon of bubble inflation. Larger microbubbles will attenuate the MR signals more effectively, but in the presence of dense microbubble clouds, their inflation will be limited by the competition for resources (e.g., gases, non-vaporized droplets) in the surroundings. Hence, more thorough investigations will be required to determine the accuracy and dynamic range of the dose–response relationships.

### 3.4. Future Applications of Nanodroplet-Mediated Dosimetry

If the discussed hurdles can be taken successfully, the proposed technology can potentially provide solutions for some of the dosimetric challenges posed by new developments in radiotherapy. For example, the expected ability to achieve dosimetric information with a high temporal resolution can be interesting in the context of FLASH radiotherapy, where conventional dosimeters typically fail due to the high dose rates used [47]. Additionally, the ability to tune the sensitivity of the nanodroplet dosimeter to different types of ionizing radiation (e.g., thermal neutrons) by varying the operation temperature can be highly useful for boron neutron capture therapy (BNCT) [38,39]. Finally, the simple use of the proposed nanodroplet-based dosimeter in combination with high-resolution read-outs using preclinical scanners might be beneficial for (preclinical) small field dosimetry, where large uncertainties on the administered doses exist [48,49].

## 4. Materials and Methods

### 4.1. Materials

All chemicals were obtained from Sigma-Aldrich (Merck, Darmstadt, Germany), unless mentioned otherwise. Perfluorobutane was purchased from F2 Chemicals Ltd. (Preston, UK) or Apollo Scientific (Manchester, UK). The 30% acrylamide/bisacrylamide (AM-BIS) solution was obtained from Bio-Rad Laboratories (Temse, Belgium). Carbopol 2050 was bought from Lubrizol (Wickliffe, OH, USA).

### 4.2. Nanodroplet Preparation

Two types of nanodroplets were used in this study. Both consisted of a perfluorobutane (PFB) core, but differed in the shell material, either lipidic 10,12-pentacosadyinoic acid (PCDA) or polymeric poly(vinyl alcohol) (PVA). A full description of the nanodroplet preparation was provided previously [11,50]. Briefly, PCDA nanodroplets were prepared by briefly fluxing PFB gas (three times 1 s) through a sealed glass vial submerged in liquid nitrogen, resulting in gas condensation. Immediately afterwards, 6 mL of the PCDA precursor solution [50] (1 mM) was added to the vial and the mixture was sonicated in an ice-cold water bath for 10 min. After sonication, 10 µL of aqueous pluronic F127 (10 mg/mL) and 0.15% *w*/*v* irgacure 2959 photoinitiator was added, before immersing the vials in an ice bath while exposing them to 352 nm UV light (UV lamp model ENF-260C, Spectroline Corporation, Melville, NY, USA) for 30 min to trigger shell polymerization. Afterwards, nanodroplets were stored in a fridge at 4 °C until further use (<1 week). Immediately before use, they were first washed, centrifugated, and resuspended in deionized water. Similarly, PVA nanodroplets were synthetized by first preparing the precursor solution. For the latter, first, 1 g of fully hydrolyzed PVA was dissolved in 50 mL deionized water and oxidized by the addition of 95 mg NaIO_4_ and subsequent stirring for 1 h. Then, glass vials were fluxed with PFB, as described before, and 5 mL of oxidized PVA solution was added. Afterwards, the mixture was sonicated for 15 min and the formed nanodroplets were allowed to continue crosslinking the shell statically in a fridge at 4 °C. After 1 h, the droplets were washed by centrifugation, resuspended in deionized water, and stored like the PCDA droplets. Previously, nanodroplet size was determined using dynamic light scattering (DLS). PCDA-PFB nanodroplets had an intensity-weighted average size of 842 ± 12 nm with a polydispersity index of 0.25 ± 0.02 and PVA-PFB nanodroplets had a diameter of 799 ± 25 nm with a polydispersity index of 0.30 ± 0.01 [21]. Finally, just prior to nanodroplet use, their concentration was determined using ^19^F NMR spectroscopy referenced against 20 mM of fluorouracil using a 400 MHz nuclear magnetic resonance (NMR) spectrometer (Bruker Biospin, Rheinstetten, Germany).

### 4.3. Phantom Preparation

To constrain the generated gas microbubbles due to radiation-induced droplet vaporization, nanodroplets were trapped in a phantom matrix. Depending on the needs of the exact experiment, different phantom containers and materials were used. For the experiment at room temperature (25 °C), nanodroplets were fixed in a gelatine matrix (3% *w*/*v*) in custom-made PVC containers. For experiment 2 in Table 1, the phantom container was replaced by a smaller version of PMMA to speed up heating after phantom preparation and the gelatine matrix was exchanged for a poly(acrylamide) hydrogel matrix [11]. The latter was prepared as follows. First, 30% (*w*/*v*) AM-BIS with a molar ratio of 29/1 was diluted to 5% (*w*/*v*) with deionized water and degassed by sonication. Then, for a phantom container of 20 mL, the following compounds were mixed: 3.25 mL of diluted AM-BIS with 16.25 mL deionized water and 0.5 mL of aqueous ammonium persulfate (8.5% *w*/*v*). Subsequently, the desired amount of nanodroplets was injected before the addition of 25 µL of TEMED to initiate polymerization for 30 min at room temperature. After gelation, any excess liquid was drained and the phantom was heated to the desired temperature in a heated water bath. To reduce the time required for phantom preparation, the phantom matrix was replaced by a carbomer solution for the final experiments at 65 °C [22,51]. For this purpose, 0.1% (*w*/*v*) carbopol 2050 was dissolved in milliQ water, the pH adjusted to 7, and the solution presaturated with PFB gas. The resulting non-Newtonian fluid allowed us to prepare the phantoms immediately at 65 °C by pouring the liquid directly in the larger PVC containers and mixing in the nanodroplets with a spatula. While the matrix still behaved as a liquid, its viscosity prevented movement of the contrast agents [52]. Finally, an ultrasound-transparent foil was attached to the top of the phantom container to prevent leakage in the water tank, while still allowing for online ultrasound read-outs. To verify survival of the nanodroplets once dispersed in the different phantom matrices, contrast generation at relevant time points was verified through acoustic droplet vaporization in preliminary experiments.

### 4.4. Irradiation Conditions

**Figure 7:**
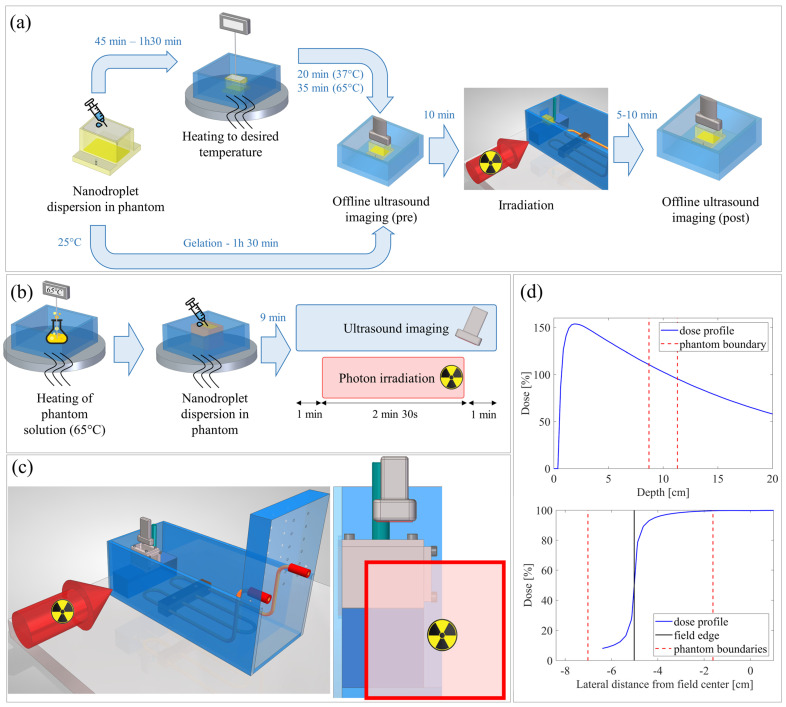
Workflow of the photon radiation experiments. Schematic representation of the experimental procedure including phantom preparation, irradiation, and offline ((**a**)—Exp. 1 and 2) or online ((**b**)—Exp. 3) ultrasound imaging. Ultrasound imaging was performed during irradiation, starting 1 min before the start of irradiation and ending 1 min after the end of irradiation. (**c**) Drawing of the radiation setup with the grey gel phantom heated in a water tank and irradiated from the side. (**d**) Dose profiles in the depth and lateral direction and how they relate with the phantom position for a field size of 10 × 10 cm^2^.

All phantom irradiations were performed in the Department of Radiotherapy at the University Hospital of Leuven, Belgium on a TrueBeam Linac (Varian Medical Systems, a Siemens Healthineers company, Palo Alto, CA, USA). A total of three different experiments were performed, assessing different aspects of the photon radiation response and ultrasound read-out. In particular, the effects of nanodroplet shell, operation temperature, beam energy, phantom matrix, and to a lesser extent, radiation and nanodroplet dose were explored, as exemplified in Table 1. Just prior to irradiation, phantoms were prepared as described before and heated in a water bath to the desired temperature. Afterwards, phantoms were transferred to the irradiation setup, consisting of a heated water tank, as depicted in Figure 7. Phantoms were irradiated from the side with the center of the phantom 10 cm deep in the water tank (Figure 7). This way, dose build-up was also covered. For the first experiment, a radiation field size of 2.4 cm (lateral) × 3 cm (height) was used, which was aligned with the center of the phantom. For the second experiment, a square field of 10 × 10 cm^2^ was positioned such that only the right half of the phantom was placed inside the radiation field to have an ‘internal control’. Similarly, for experiment 3, a 10 × 10 cm^2^ field was used, but this time only the lower right quadrant of the phantoms was irradiated. Control phantoms for the different conditions underwent the same procedure, but instead of being irradiated in the dedicated water tank, they were kept in a control water bath at the same temperature for a similar duration as the irradiation. In addition, each naked phantom matrix (i.e., without nanodroplets) was irradiated to verify the absence of radiation response due to the gel composition itself.

### 4.5. Ultrasound Acquisition

Ultrasound images were acquired using the DiPhAS experimental ultrasound scanner (Fraunhofer IBMT, Sulzbach (Saar), Germany), driving a 7.5 MHz L7-XTech probe (Vermon, Tours, France). Plane wave imaging was employed with compounding of five different angles. In the first two experiments, phantoms were only imaged offline pre- and post-irradiation. To cover the entire phantom, the ultrasound probe was fixed on a linear stage and moved along the beam direction (i.e., phantom images were acquired perpendicular to the beam direction). This resulted in 20–25 frames per phantom. For the final experiment, ultrasound imaging was performed online during irradiation. For this purpose, the ultrasound scanner was placed inside the treatment bunker and the probe was fixed on a frame with a motorized stage. The latter was programmed such that the probe was only submerged in the water tank during imaging/irradiation, to prevent heat damage at 65 °C. Additionally, the scanner was placed as far as possible from the Linac head, to prevent any damage due to (scatter) dose (Figure 7). Image acquisition was performed at a frame rate of 10 Hz and was longer than the actual irradiation to provide sufficient buffer time to secure the room and start the irradiation remotely.

### 4.6. Image Processing

Ultrasound images were processed in MATLAB (MathWorks, Natick, MA, USA). For the first and second experiment, beamformed data were visualized by performing a Hilbert transform followed by log compression to dB scale. The radiation response was further quantified by counting individual microbubbles in dedicated ROIs. Microbubbles were identified based on an empirical gray value threshold, whereby we assumed that the brightest pixel identifies the center of the microbubble. Double counting was prevented by considering centers that were located too close to one another (empirical near neighbor approach) as stemming from the same microbubble. The resulting identification of individual microbubbles is illustrated in Figure 3. For the third experiment, vaporization events were also counted, but a different approach was used, as bubble densities were much larger. First, raw beamformed data were interpolated to achieve a uniform pixel density in each dimension. Then, differential images were calculated by subtracting subsequent frames. Afterwards, a Wiener filter was applied to smoothen the data and log compression to dB scale was performed. Finally, vaporization events were detected using empirical thresholding and localized by determining the weighted centroid of the point spread function [53] (regionprops function).

### 4.7. Magnetic Resonance Imaging Contrast

Finally, we investigated the MR contrast induced by the phase-change ultrasound contrast agents. For this purpose, various concentrations of PVA-PFB nanodroplets were dispersed in gelatine (2% *w*/*v*) phantoms, as described before. In this experiment, droplet vaporization was triggered using high-pressure ultrasound waves from a Vevo2100 ultrasound scanner (Fujifilm VisualSonics, Toronto, ON, Canada). Only half of the phantom was exposed to allow quantification of the drop in MR signal intensity after droplet vaporization. MR images were acquired on a 9.4T MR scanner (Bruker, Kontich, Belgium) using a custom-built ^19^F/^1^H coil. A TurboRARE sequence was employed using the parameters listed in Table 2.

## Figures and Tables

**Table 1:** Overview of the different tests performed to examine the nanodroplet photon radiation response.

Exp.	Matrix	Amount of Phantoms	ND Shell	ND Conc. [µM ^19^F]	Temp. [°C]	Dose [Gy]	Dose Rate [Gy/min]	Energy [MV]	US Imaging
1	Gelatine	1	-	-	25	10	2	10	Offline
		2	PCDA	25	25	-	-	-	
		3	PCDA	25	25	10	2	10	
		2	PVA	25	25	-	-	-	
		3	PVA	25	25	10	2	10	
2	poly(acryl-amide) gel	2	PVA	50	37	-	-	-	Offline
		2	PVA	50	37	10	4	6	
		2	PVA	50	37	10	4	15	
		1	-	-	65	4	4	6	
		3	PVA	25	65	-	-	-	
		5	PVA	25	65	10	4	6	
3	Carbopol	1	-	-	65	4	4	6	Online
		1	PVA	40	65	2	0.8	6	
		6	PVA	20	65	6	2.4	6	

**Table 2:** MRI acquisition parameters.

Parameter	Quantity	Parameter	Quantity
Echo time	27.41 ms	Matrix size	256 × 256
Repetition time	2 s	Field of view	45 × 35 mm
Number of averages	4	Scan time	4 min 16 s
RARE factor	8		

## Data Availability

Data is contained within the article and Supplementary Material.

## Supplementary Materials

The following supporting information can be downloaded at: https://www.mdpi.com/article/10.3390/ph17050629/s1. Figure S1: Ultrasound contrast generation of PVA-PFB nanodroplets after a radiation and acoustic stimulus. Gelatine phantom containing 25 µM of PVA-PFB nanodroplets before (left) and after (middle) irradiation with 10 Gy photons at 2 Gy/min, and after subsequent acoustic droplet vaporization (ADV, right). Figure S2: Offline stacking of 2D ultrasound images into a 3D dataset using ImageJ and 3D Slicer.

## Appendix A

Theory of radiation-induced nucleation of superheated emulsions:

(A1) $$R_{c}=\frac{2\sigma }{\left(p_{s}-p_{l})(1-\frac{\rho_{v}}{\rho_{l}}\right)}$$

(A2) $$W_{tot}=\frac{16\pi \sigma^{3}}{3{\left(p_{s}-p_{l}\right)}^{2}{\left(1-\frac{\rho_{v}}{\rho_{l}}\right)}^{2}}\times \left[1+\frac{2∆H}{{(p}_{s}-p_{l})(\frac{1}{\rho_{v}}-\frac{1}{\rho_{l}})}-3\frac{T}{\sigma }\frac{d\sigma }{dT}\right]+W_{irr}$$

(A3) $$W_{irr}=2\pi \rho_{l}R_{c}^{3}\dot{R^{2}}$$

(A4) $$\dot{R}=\frac{4D{\left(\frac{\rho_{l}}{\rho_{v}}\right)}^{1/3}}{R_{c}}$$

(A5) $$D=\frac{k}{\rho_{l}c_{p}}$$

(A6) $$Vt=\frac{W_{tot}}{aR_{c}}$$

(A7) $$s=\frac{T-T_{b}}{T_{c}-T_{b}}$$

with *R_c_* the critical radius and all other quantities explained in Table A1.

**Table A1:** Thermodynamic properties and corresponding calculated vaporization thresholds of various perfluorocarbons: perfluorobutane (PFB), heptafluoropropane (HFP), and octafluoropropane (OFP) [18,19,54,55,56].

Quantity	PFB			HFP	OFP
Temperature (T, [K])	298	310	338	310	310
Surface tension (σ, [N m^−1^])	7.19 × 10^−3^	6.04 × 10^−3^	3.48 × 10^−3^	5.85 × 10^−3^	2.39 × 10^−3^
Differential surface tension (dσ, [N m^−1^])	−9.90 × 10^−4^	−1.91 × 10^−3^	−8.80 × 10^−4^	−1.01 × 10^−3^	−6.10 × 10^−4^
Differential temperature (dT, [K])	10.0	20.0	10.0	9.00	5.00
Saturation pressure (p_s_, [Pa])	2.68 × 10^5^	3.88 × 10^5^	8.24 × 10^5^	6.62 × 10^5^	1.15 × 10^6^
Liquid pressure (p_l_, [Pa])	1.01 × 10^5^	1.01 × 10^5^	1.01 × 10^5^	1.01 × 10^5^	1.01 × 10^5^
Heat conductivity (k, [W m^−1^ K^−1^])	4.27 × 10^−2^	4.10 × 10^−2^	3.67 × 10^−2^	5.51 × 10^−2^	3.67 × 10^−2^
Gas density (ρ_v_, [kg m^−3^])	28.9	41.7	66.7	53.0	121
Liquid density (ρ_l_, [kg m^−3^])	1.50 × 10^3^	1.45 × 10^3^	1.34 × 10^3^	1.34 × 10^3^	1.26 × 10^3^
Specific heat capacity (c_p_, [J kg^−1^ K^−1^])	1.08 × 10^3^	1.11 × 10^3^	1.18 × 10^3^	1.30 × 10^3^	1.26 × 10^3^
Latent vaporization heat (ΔH, [J kg^−1^])	8.75 × 10^4 †^			1.04 × 10^5^	6.22 × 10^4^
Boiling temperature (T_b_, [K])	272			257	236
Critical temperature (T_c_, [K])	386			375	345
Nucleation parameter (a, [-])	2.00	2.00	2.00	2.00	2.00
Vaporization threshold (Vt, [keV/µm])	369	147	21.8	69.0	11.1
Reduced superheat (s, [-])	0.232	0.336	0.580	0.452	0.679

^†^ Not temperature-independent, but conflicting values have been described by NIST, so we opted for a representative average.
