# Supplementary material for: A Preliminary Investigation of Radiation-Sensitive Ultrasound Contrast Agents for Photon Dosimetry
Source: Pharmaceuticals (Basel). 2024 May 14;17(5):629. doi: 10.3390/ph17050629 (PMC11125270; doi:10.3390/ph17050629)
Supplement: Supplementary file 1 [file pharmaceuticals-17-00629-s001.zip › pharmaceuticals-2950444-supplementary.pdf]

A preliminary investigation of radiation-sensitive ultrasound contrast agents for photon dosimetry

Bram Carlier*, Sophie V. Heymans*, Sjoerd Nooijens, Gonzalo Collado-Lara, Yosra Toumia, Laurence Delombaerde, Gaio Paradossi, Jan D'hooge, Koen Van Den Abeele, Edmond Sterpin, Uwe Himmelreich

Supplementary material

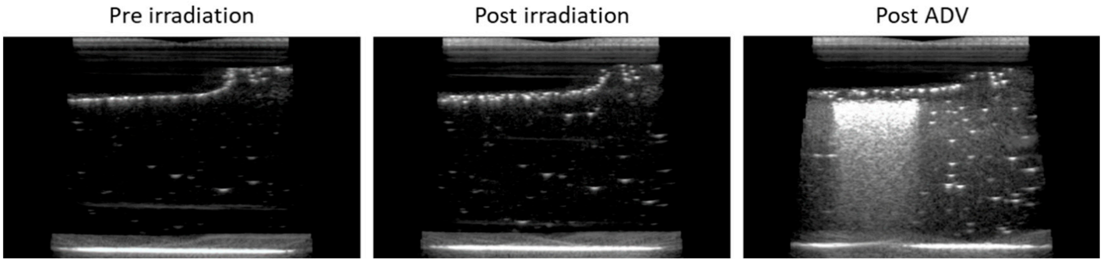

Figure S1. Ultrasound contrast generation of PVA-PFB nanodroplets after a radiation and acoustic stimulus. Gelatine phantom containing 25 µM of PVA-PFB nanodroplets before (left) and after (middle) irradiation with 10 Gy photons at 2 Gy/min, and after subsequent acoustic droplet vaporization (ADV, right).

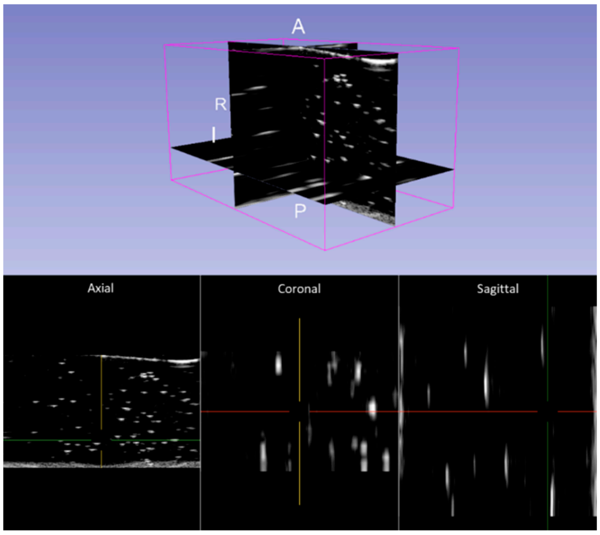

Figure S2. Offline stacking of 2D ultrasound images into a 3D dataset using ImageJ and 3D slicer.
